# Evaluation of Passive Samplers as a Monitoring Tool for Early Warning of *Dinophysis* Toxins in Shellfish

**DOI:** 10.3390/md11103823

**Published:** 2013-10-11

**Authors:** Gemita Pizarro, Ángeles Moroño, Beatriz Paz, José M. Franco, Yolanda Pazos, Beatriz Reguera

**Affiliations:** 1 Spanish Institute of Oceanography (IEO), Oceanographic Centre of Vigo, Subida a Radio Faro 50, Vigo 36390, Spain; E-Mails: gemita.pizarro@ifop.cl (G.P.); beapaz@uvigo.es (B.P.); 2 Technological Institute for the Control of the Marine Environment of Galicia (INTECMAR), Peirao de Vilaxoán s/n, Vilagarcía de Arousa, Pontevedra 36611, Spain; E-Mails: amorono@intecmar.org (Á.M.); ypazos@intecmar.org (Y.P.); 3 Institute of Marine Research (CSIC), Eduardo Cabello 6, Vigo 36080, Spain; E-Mail: jose.franco@vi.ieo.es

**Keywords:** *Dinophysis*, early warning DSP outbreaks, HAB monitoring, modeling toxin accumulation, Solid Phase Adsorbing Toxin Tracking (SPATT), Galician Rías

## Abstract

From June 2006 to January 2007 passive samplers (solid phase adsorbing toxin tracking, SPATT) were tested as a monitoring tool with weekly monitoring of phytoplankton and toxin content (liquid chromatography–mass spectrometry, LC-MS) in picked cells of *Dinophysis* and plankton concentrates. Successive blooms of *Dinophysis acuminata*, *D. acuta* and *D. caudata* in 2006 caused a long mussel harvesting closure (4.5 months) in the Galician Rías (NW Spain) and a record (up to 9246 ng·g resin-week^−1^) accumulation of toxins in SPATT discs. Best fit of a toxin accumulation model was between toxin accumulation in SPATT and the product of cell densities by a constant value, for each species of *Dinophysis*, of toxin content (average) in picked cells. Detection of *Dinophysis* populations provided earlier warning of oncoming diarrhetic shellfish poisoning (DSP) outbreaks than the SPATT, which at times overestimated the expected toxin levels in shellfish because: (i) SPATT accumulated toxins did not include biotransformation and depuration loss terms and (ii) accumulation of toxins not available to mussels continued for weeks after *Dinophysis* cells were undetectable and mussels were toxin-free. SPATT may be a valuable environmental monitoring and research tool for toxin dynamics, in particular in areas with no aquaculture, but does not provide a practical gain for early warning of DSP outbreaks.

## 1. Introduction

Dinoflagellate species of the genus *Dinophysis* have two kinds of lipophilic shellfish toxins (LST): diarrhetic shellfish poisoning (DSP) toxins—okadaic acid (OA) and dinophysistoxins (DTXs)—and pectenotoxins (PTXs) [1]. A few hundred cells of *Dinophysis* per liter, difficult to monitor due to their very patchy distribution, may lead to accumulation of toxins in shellfish [2,3,4] above regulatory levels established by the European Union [5] and lead to lengthy shellfish harvesting closures. The chronic detection of lipophilic shellfish toxins (OA, DTXs, PTXs) above regulatory levels in shellfish is a major threat to shellfish resources in Atlantic coastal waters of Europe [6]. To date, 12 species of *Dinophysis* have been found to contain lipophilic toxins, and blooms of seven of these have been associated with toxic outbreaks [7]. Large differences in toxin profile and content have been found between strains of the same species from different locations [6,8]. Further, the toxin per cell of the same species in the same location may vary up to one order of magnitude throughout its growing season [9]. For all these reasons, the use of “trigger levels” of *Dinophysis* cells as a monitoring practice to start shellfish analyses or even to ban bivalve harvesting cannot be recommended [4,6,10].

MacKenzie *et al*. [11] introduced the use of passive samplers as a tool for early detection of toxins in aquaculture sites; these are made of porous synthetic resins able to adsorb lipophilic substances, including lipophilic shellfish toxins (LST). These authors claimed that during proliferations of *Dinophysis* in New Zealand coastal waters, an important proportion of their toxins were released into the seawater; they suggested that “solid phase adsorption toxin tracking” (SPATT) mesh bags filled with the porous resins (SPATT discs herein) could be used as an early warning tool in toxin monitoring programmes. SPATT discs have been tested at different shellfish growing sites in Europe, but for brief periods of time, and their performance has been compared with that of an artificial mussel, with the advantage that toxin extraction from the resins is easier and avoids interferences in chromatographic analyses due to complex matrices found in shellfish meat extracts [9,12,13,14].

**Figure 1:**
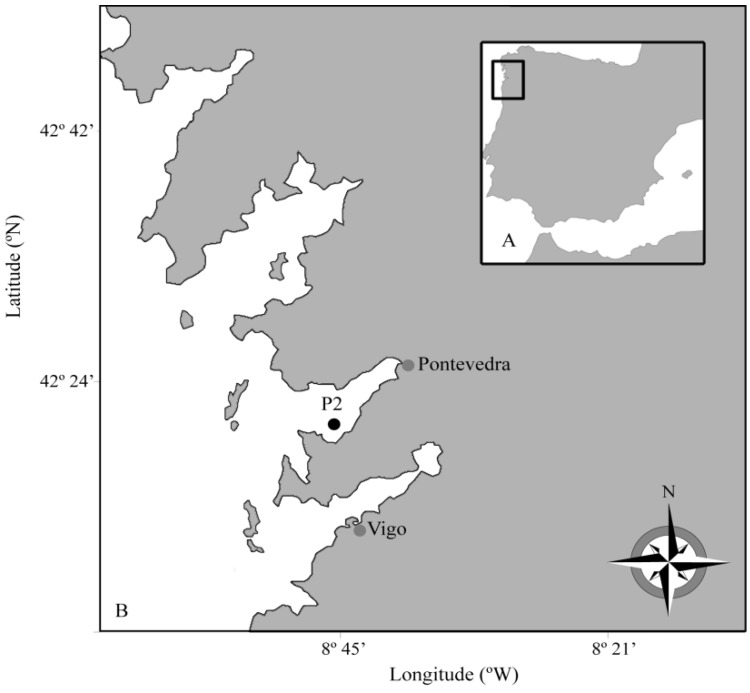
(**a**) The Galician Rías Baixas (**b**) The study area and location of the P2 station in Ría de Pontevedra.

During 2006, SPATT discs were deployed at different depths in the water column next to a mussel cultivation raft and renewed at weekly intervals during the whole growing season of *Dinophysis* spp. The study was carried out at a fixed station in Ría de Pontevedra, one of the four Galician Rías Baixas (NW Spain), site of intensive mussel production (Figure 1). This region suffers from chronic blooms of several species of *Dinophysis*, and DSP outbreaks lead to lengthy shellfish harvesting closures every year. The objective of this work was to evaluate the use of *in situ* deployed SPATT discs as a tool for (i) early detection of *Dinophysis* and its toxins in aquaculture sites with established molluscan shellfish safety monitoring programs; (ii) studying the *in situ* toxin dynamics associated with *Dinophysis* blooms.

## 2. Results

### 2.1. Seasonal Distribution of *Dinophysis* Species

**Figure 2:**
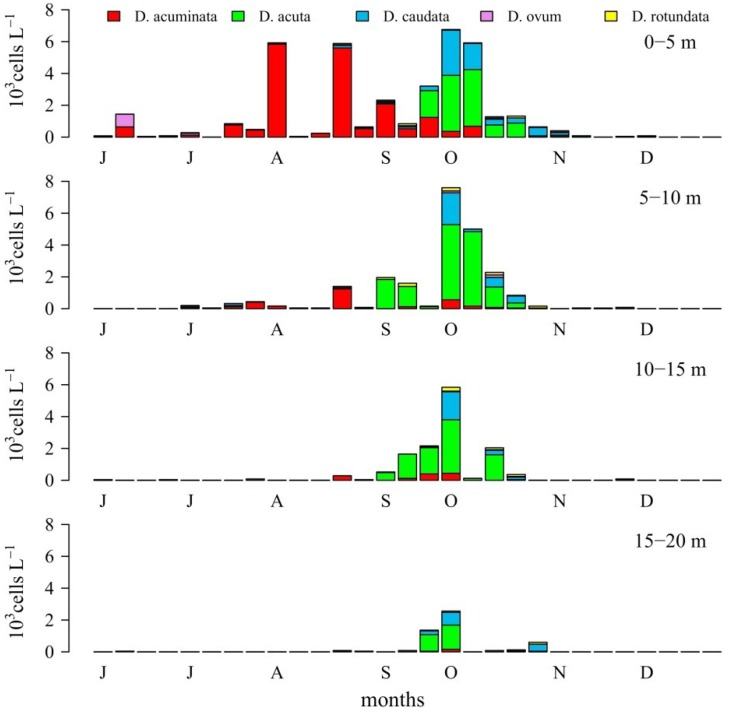
Weekly distribution of *Dinophysis* species from depth integrated water column samples, at 0–5 m, 5–10 m, 10–15 m and 15–20 m, collected at the P2 station in Ría de Pontevedra.

Weekly monitoring estimates from integrated water column samples (0–5, 5–10, 10–15, 15–20 m) showed that in summer–autumn 2006, the first short-lived *Dinophysis* peak of the season (co-occurring with *D. acuminata*) on 12 June corresponded to a “*Dinophysis* sp.” later identified as *D. ovum* [15]. From then until late August, *D. acuminata*, with cell maxima most times at 0–5 m, was the overwhelmingly dominant species of *Dinophysis*. From 28 August to 24 October *D. acuta* became dominant while *D. acuminata* densities progressively declined. *D. caudata* densities started to increase on 25 September and its cell maxima co-occurred with those of *D. acuta* until the end of October. Both *D. acuta* and *D. caudata* showed a more even distribution in the 0–15 m water column, and their cell maxima appeared most times at 5–10 m and 10–15 m. Finally by mid-November a sudden mono-specific peak of *D. caudata* was observed at 0–5 m (Figure 2).

### 2.2. *Dinophysis* spp. in the Concentrated (Pump) Phytoplankton Samples

**Figure 3:**
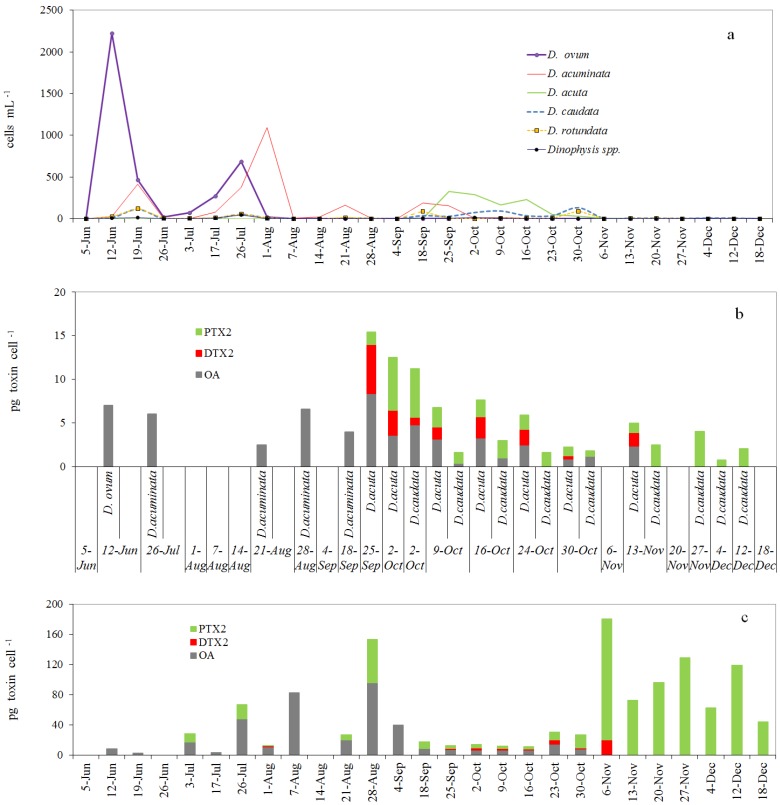
*Dinophysis* species from pump concentrates collected at station P2 (Ría de Pontevedra), between June and December 2006, at a fixed depth in the top 5 m (**a**) Distribution of cell densities of different species of *Dinophysis*; (**b**) Estimates of toxin content (pg cell^−1^) in single-cell isolates (picked cells) (**c**) Estimates of average toxin content (pg cell^−1^) per cell of *Dinophysis* in pump concentrates.

Distribution of *Dinophysis* species in the pump concentrates showed a similar succession to that observed on the 0–5 m section of the integrated water column samples. An exception was the occurrence and dominance of *D. ovum*, which was practically the only species of *Dinophysis* in the size-fractioned (77–20 µm) plankton concentrate on 12 June and the dominant species of *Dinophysis* until 26 July. In contrast *D. acuminata* was reported as the dominant species in the 0–5 m hose-samples from 17 July to mid-September. *D. rotundata* (=*Phalacroma rotundatum*) was also present (not included in the figure) representing a very small percentage of the total community of *Dinophysis* species (Figure 3a).

### 2.3. Toxin Profile and Content in Picked Cells of *Dinophysis* from the Pump Concentrates

Okadaic acid (OA) was the only toxin detected in picked cells of *D. ovum* (1.4–7.0 pg cell^−1^) and *D. acuminata* (1.7–6.6 pg cell^−1^) (Figure 3b). *D. acuta* was the species with the highest toxin content per cell. Its toxin profile was always dominated by OA (0.9–8.4 pg cell^−1^), followed by DTX2 (0.4–5.6 pg cell^−1^) and PTX2 (1.0–6.1 pg cell^−1^). *D. caudata* showed a more variable profile: PTX2 (0.6–5.5 pg cell^−1^) was the dominant toxin or even the only toxin present in some samples; OA (nd–4.8 pg cell^−1^) was detected in three out of nine samples and traces of DTX2 (0.9 pg cell^−1^) in only one sample where OA and DTX2 were predominant.

### 2.4. Toxin Profiles and Content per Cell of *Dinophysis* in the Size-Fractioned Plankton Concentrates

OA, DTX2 and PTX2 were the predominant toxins in the size-fractioned (77–20 µm) phytoplankton pump-concentrates (Figure 3c). OA was the predominant toxin in concentrates rich in *D. ovum* and *D. acuminata* between 12 June and 7 August. After this period and until 18 September, the predominance of OA was associated with *D. acuminata*. The presence of PTX2 within this same period (3 July–18 September), and traces of DTX2 on 1 August corresponded to the occurrence of moderate densities of *D. caudata*. From 25 September to 30 October OA, followed by PTX2 and DTX2 were the predominant toxins in the *D. acuta-*dominated plankton concentrates. The PTX2 increase observed in the toxin profiles on 30 October co-occurred with a new increase of *D. caudata*. 

After 6 November and until 18 December PTX2 was the only toxin detected in the plankton concentrates when *Dinophysis* dropped to densities (<9 cells mL^−1^) close to detection levels. 

Estimates of toxin per cell from pump concentrates were often much higher (even an order of magnitude) than those obtained from picked cells. Thus, maximum values of 80.4 pg OA cell^−1^ were observed during the bloom of *D. acuminata* by the end of August and of 161 pg PTX2 cell^−1^ by the end of the bloom of *D. acuta* plus *D. caudata* in early November.

### 2.5. Weekly Adsorption of Toxins in the SPATT Discs and Its Relation with *Dinophysis* Populations

**Figure 4:**
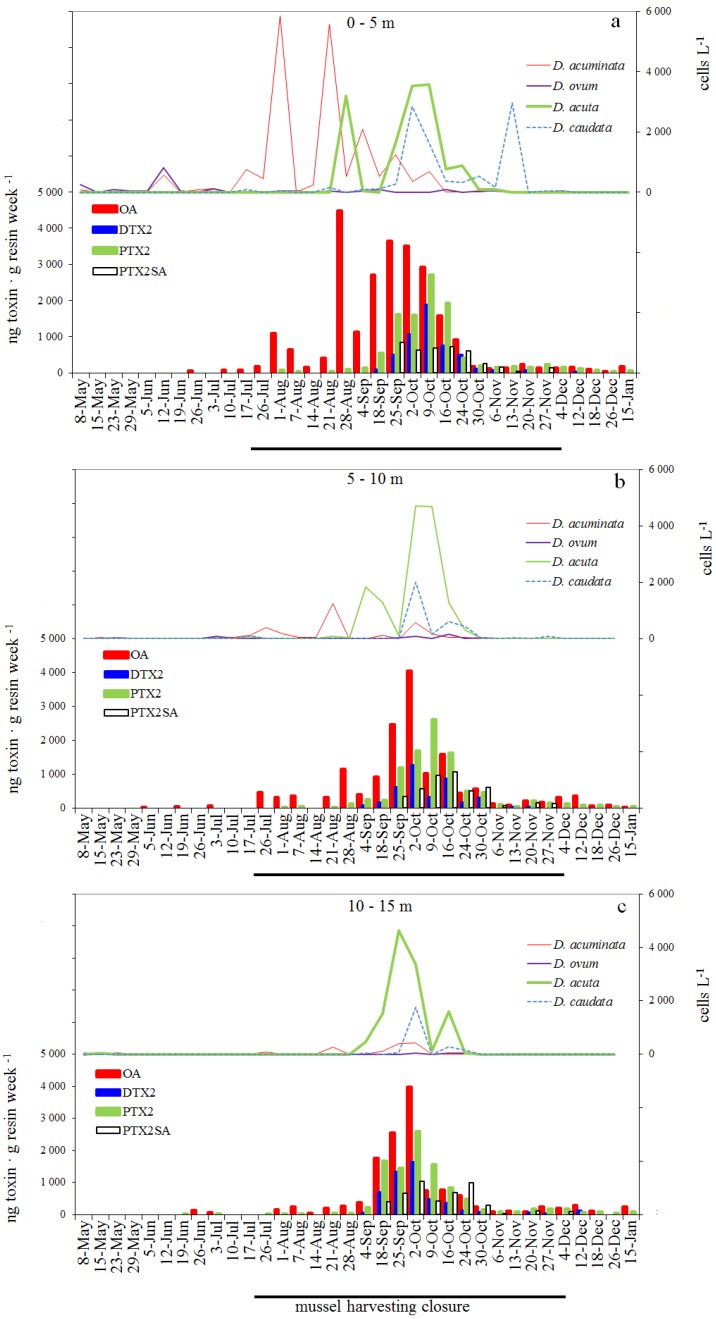
Distribution of *Dinophysis* (integrated water column) and weekly adsorption of toxins by the SPATT discs (ng toxin·g resin·week^−1^) (**a**) *Dinophysis* at 0–5 m and SPATT discs at 3 m (**b**) *Dinophysis* at 5–10 m and SPATT discs at 7 m (**c**) *Dinophysis* at 10–15 m and SPATT discs at 12 m.

Weekly adsorption of toxins in the SPATT discs included those detected in picked cells of *Dinophysis* and in the plankton concentrates—OA, DTX2, PTX2—plus PTXSA, a toxin known to result from enzymatic transformation of PTX2 [16,17] (Figure 4).

#### 2.5.1. *SPATT at 3 m* versus Dinophysis *Cells at 0–5 m*

OA adsorbed by the SPATT discs deployed at 3 m ranged between 66 and 4495 ng·g resin-week^−1^. There was a lag of 2 weeks between the first peaks (>10^3^ cells·L^−1^) of *D. ovum* + *D. acuminata* (12 June) and the first detection of OA (26 June) by the SPATT discs (Figure 4a). The highest adsorption (4495 ng·g resin-week^−1^) on 28 August was observed following the seasonal maximum of *D. acuminata* (10^4^ cell·L^−^^1^) the previous week. New high levels of OA (up to 3640 ng·g resin-week^−1^) were observed between 28 August and 10 October when *D. acuta* and *D. acuminata* co-occurred. After mid October concentrations gradually declined (<200 ng·g resin-week^−1^) until the end of the study. High densities of *D. acuta* on 2–9 October with a moderate toxin per cell did not contribute to substantial accumulation of OA the following days.

DTX2 accumulated by the SPATT discs ranged between 30 and 1876 ng·g resin-week^−1^. There was a lag of 21 days between the onset of the *D. acuta* bloom (28 August) and the adsorption of DTX2 by the SPATT (18 September). The highest accumulation (1876 ng·g resin·week^−1^) on 9 October followed the peaks of *D. acuta* and *D. caudata* the previous week, although DTX2 content in the former species was threefold that of the latter (Figure 3b), so the contribution of *D. caudata* may not have been significant. After 9 October, DTX2 was no longer detected in picked cells of *D. caudata* cells, so this toxin was contributed only by the population of *D. acuta*. DTX2 was still found in the SPATT discs 7 days after *D. acuta* was no longer detected.

PTX2 adsorption ranged between 40 and 2705 ng·g resin-week^−1^. This toxin was first found on 1 August, *i.e.*, 15 days after detection of 80 cells·L^−1^ of *D. caudata* at 10–15 m. PTX2 was first detected in the phytoplankton pump-concentrates on 23 and 26 of July, and was tracked by the SPATT discs at 3 m until the end of the study, almost 2 months after *Dinophysis* species were below detection levels.

Concentrations of PTX2SA (37 to 845 ng·g resin-week^−1^), a toxin that was never detected either in picked cells of *Dinophysis* or in the pump concentrates, was significantly related (*r*^2^ = 0.78, *p* < 0.001) with those of PTX2 at the same depth.

#### 2.5.2. *SPATT Discs at 7 m* versus Dinophysis *Cells at 5–10 m*

The ranges of OA, DTX2, PTX2 and PTXSA adsorbed by the SPATT discs at 7 m were 22–4053, 30–1264, 19–1690 and 72–1072 ng·g resin-week^−1^, respectively (Figure 4b).

OA was first tracked by the SPATT discs on 5 and 19 June when *D. acuta* had not yet been detected and very low densities of *D. acuminata* and *D. caudata* were found in this layer of the water column. As in the 3 m SPATT, OA was tracked until the end of the study period.

DTX2 was not detected in the SPATT discs either when a slight increase of *D. acuta* and *D.* *caudata* was observed on 17 July or when there was a new increase of *D. acuta* on 21 August. Maximal concentrations (up to 1264 ng·g resin-week^−1^) on 2 and 16 October were found just before and after the seasonal maxima of *D. acuta*. DTX2 were last detected at this depth on 24 October, *i.e.*, 21 d after *D. acuta* was no longer detected in the plankton samples.

PTX2 was detected 15 days after the increase of *D. acuta* and *D. caudata* densities on 17 July. After this date, both species went undetected, but the SPATT discs continued tracking PTX2. *D. acuta* reappeared on 21 August and lasted until 24 October, and *D. caudata* was detected again on 2 October and dropped to undetectable levels on 27 November. However PTX2 was still present in the SPATT discs nearly 2 months later.

As in the 3 m SPATT discs, PTX2SA was significantly related (*r*^2^ = 0.80, *p* < 0.001) with the concentrations of PTX2 adsorbed at the same depth.

#### 2.5.3. *SPATT Discs at 12 m* versus Dinophysis *Cells at 10–15 m*

OA (49–3994 ng·g resin-week^−1^) was tracked on 26 June and 3 July when *Dinophysis* cells fell below the detection limit at 10–15 m (Figure 4c). The highest adsorption of this toxin (3994 ng·g resin-week^−1^) was observed on 2 October, *i.e.*, 7 days after the *D. acuta* maximum was observed. Although *Dinophysis* cells were no longer observed at 10–15 m after 3 October, OA was still found in the SPATT discs until the end of the study.

DTX2 (42–1634 ng·g resin-week^−1^) was first found in the 12 m SPATT discs on 4 September coinciding with a sustained increase of *D. acuta*. Maximum adsorption of DTX2 (1634 ng·g resin-week^−1^) on 2 October was 7 days after the seasonal maximum of *D. acuta*. Although *D. acuta* and *D. caudata* had fallen to undetectable levels since 24 October, DTX2 was tracked until 12 December.

PTX2 (5–2590 ng·g resin-week^−1^) maximal adsorption was observed on 2 October, 7 days after the peak value of *D. acuta* (4640 cell·L^−^^1^). Moderate levels of this toxin were found in the SPATT discs until the end of the study.

PTX2SA (32–1028 ng·disc^−^^1^) was significantly related (*r*^2^ = 0.65, *p* < 0.001) with concentrations of PTX2 found at the same depth.

### 2.6. Simulations of Toxin Adsorption by SPATT at 3 m, 7 m and 12 m

**Figure 5:**
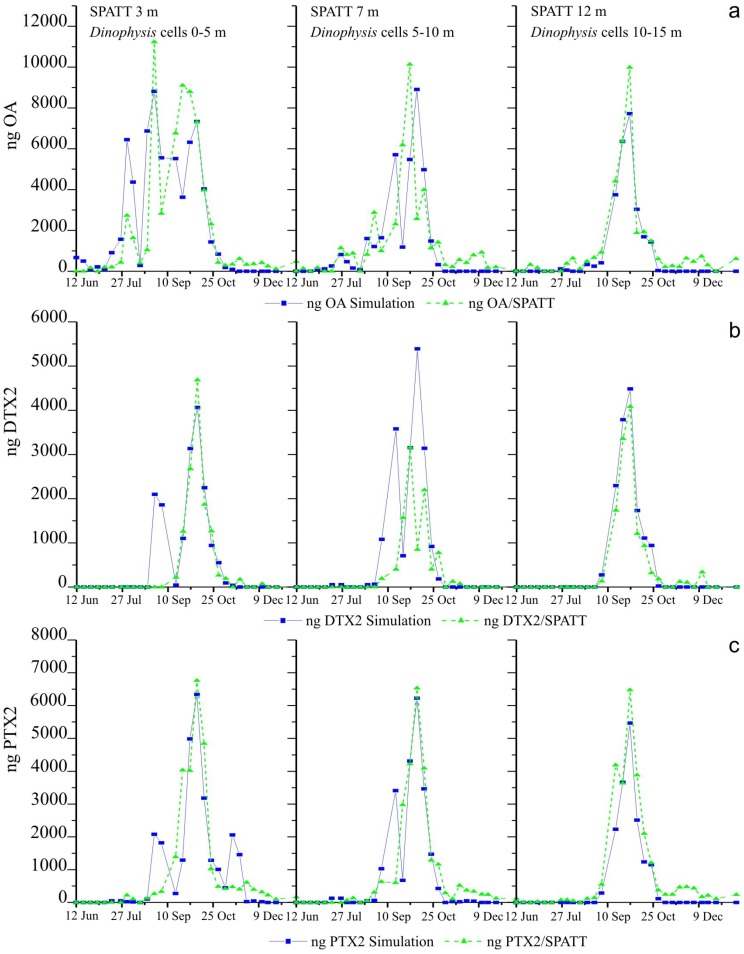
Simulated fits of toxin accumulation on the SPATT based on the average of toxin content (pg cell^−1^) per cell of *Dinophysis* estimated from analyses of picked cells of each species, to data of accumulation of (**a**) okadaic acid (OA) (**b**) dinophysistoxins (DTX2) (**c**) pectenotoxins (PTX2).

Simulation based on toxin content of picked cells of *Dinophysis* provided a good fit to the SPATT deployed at 12 m, quite good fit to the SPATT at 3 m, and for PTX2 to the SPATT at 7 m. Fits for OA and DTX2 to the SPATT at 7 m were not so good, although the correlation between simulated and actual data was always statistically significant (*p* < 0.005) (Figure 5, Table 1). Model concentrations of all toxins accumulated on the SPATT reached were very close to the actual data (Figure 5). In contrast most of the fits based on toxin per cell estimated from the size-fractioned plankton concentrates were not good, although they were statistically significant in most cases (*p* < 0.005) with the exception of the PTX2 toxin on the SPATT at 3 m and the OA on the SPATT at 12 m.

**Table 1:** Main parameters of the linear regressions between the actual data of toxins accumulated by SPATT and the simulation based on *Dinophysis* field concentrations (cell·L^−1^) and their toxin content (pg cell^−1^) estimated from liquid chromatography–mass spectrometry (LC-MS) analyses of picked cells and pumped size-fractioned (77–20 µm) plankton concentrates (plankton).

Toxin	Source of toxin data	SPATT, 3 m		SPATT, 7 m		SPATT, 12 m	
	(pg cell^−1^)	*r*^2^	slope	*r*^2^	slope	*r*^2^	slope
OA	picked cells	0.62	0.70	0.34	0.61	0.95	0.87
	plankton	0.44	1.74	0.07	1.35	0.68	1.96
DTX2	picked cells	0.74	0.90	0.39	1.18	0.98	1.14
	plankton	0.46	0.82	0.44	1.16	0.78	0.92
PTX2	picked cells	0.72	0.78	0.79	0.88	0.94	0.79
	plankton	0.19	1.13	0.65	1.70	0.90	1.75

**Figure 6:**
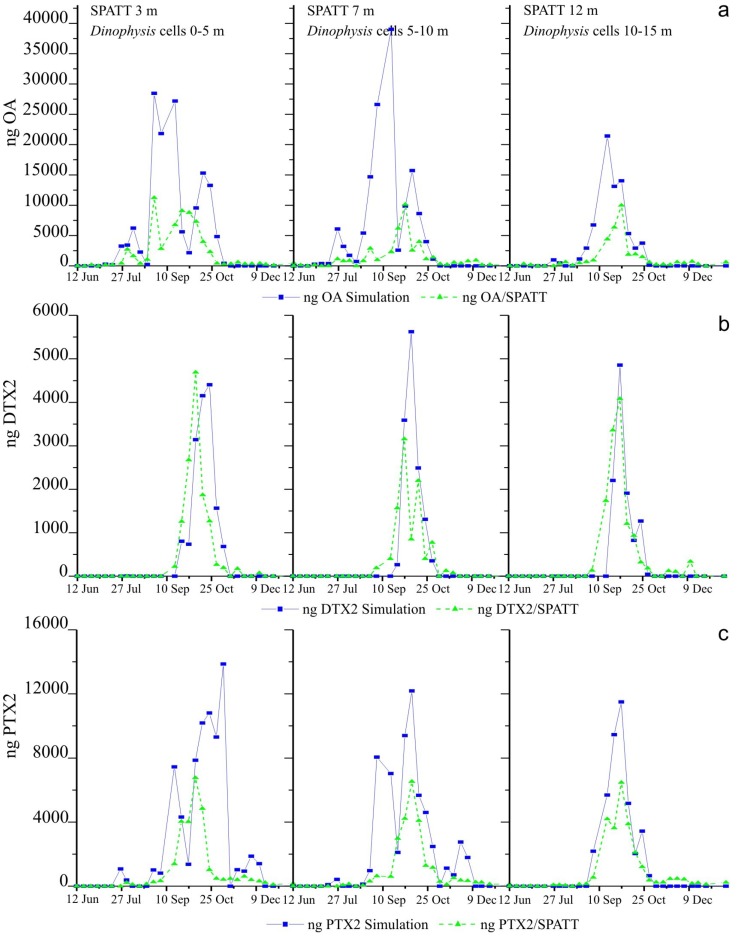
Simulated fits of toxin accumulation on the SPATT, based on toxin content (pg cell^−1^) per cell of *Dinophysis* estimated from analyses of the size-fractioned (77–20 µm) plankton concentrates, to the actual data of accumulation (**a**) OA (**b**) DTX2 (**c**) PTX2.

Concentrations of accumulated toxins in this case largely overestimated the real amounts found in the SPATT discs except in the case of DTX2, a toxin for which simulated and actual data were very close (Figure 6).

## 3. Discussion

### 3.1. Seasonal and Annual Variability of the Toxin Profile and Content in Picked Cell of *Dinophysis*

Knowledge on the toxin profile and content of *Dinophysis* species contributing to toxic outbreaks in a given region are essential parameters for predictive models on toxin uptake and depuration in shellfish. Nevertheless the toxic potential of each species may show considerable seasonal [9] and interannual variability and needs to be frequently evaluated. Table 2 compiles data on lipophilic toxin profile and content of *Dinophysis* cells from previous studies in the Galician Rías (Pontevedra and Vigo) in addition to the present study. All toxin analyses were performed by LC-MS under identical conditions to those mentioned in Section 4.3 and Section 4.4 for the systematic sampling conducted during 2006. These results confirm the presence of only OA at detectable levels in the toxin profile of *D*. *acuminata*, and show important interannual differences. For example, the average toxin content of *D. acuminata* specimens isolated in 2006 was threefold that observed in those from 2005. OA, DTX2 and PTX2 were always observed in the toxin profile of *D. acuta* in the 23 samples analyzed during 2005 and 2006. The interannual differences in toxin content of this species were not as marked as in the case of *D. acuminata.* PTX2 was the dominant or even the only toxin present in the profile of *D. caudata*. This species showed considerable changes in both toxin profile (OA and/or DTX2 in addition to PTX2) and content. *D. rotundata* (=*Phalacroma rotundatum*) specimens were either non toxic, or exhibited a very low toxin content (<1 pg cell^−1^) with profiles common to those present in co-occuring species of *Dinophysis*. An earlier study [18] asked whether this heterotrophic species produces toxins *de novo* or just takes it up from its ciliate prey previously fed with toxic *Dinophysis* spp.

**Table 2:** Interannual variability of toxin content (pg cell^−1^) in picked cells of different species of *Dinophysis* from Ría de Pontevedra (station P2) and Ría de Vigo (Stations D13, Moaña), NW Spain, analyzed with identical methods.

Cellular toxin content (pg cell^−1^), mean ± SD							
*Dinophysis* species	year	OA	DTX2	PTX2	*n*	Location	Reference
*D. acuminata*	2002	12.1	nd	nd	1	Bueu	[19]
	2003	1.0	nd	nd	1	Bueu	[19]
	2005	1.3 ± 1.0	nd	nd	15	Bueu	[19]
	2006	3.7 ± 1.8	nd	nd	8	Bueu	this work
*D. skagii*	2006	nd	nd	nd	1	Bueu	this work
*D. acuta*	2005	3.8 ± 2.5	3.7 ± 2.4	1.5 ± 1.0	11	Bueu	[19]
	2006	2.9 ± 2.0	1.9 ± 1.5	2.8 ± 2.7	12	Bueu	this work
*D. caudata*	2003	nd	nd	44.4 ± 15.1	2	Bueu	[19]
	2005	nd	nd	3.9	1	D13	[19]
	2006	0.6	2.8	5.0	1	Moaña	this work
*D. rotundata*	2003	0.2 ± 0.2	nd	0.3 ± 0.4	5	Bueu	[18]
	2005	0.4 ± 0.5	0.5 ± 0.6	nd	2	Bueu	[18]
	2007	nd	nd	nd	4	Bueu	[18]

### 3.2. Simulations of Toxin Accumulation in the SPATT *versus* the Actual Observations: What Is the Source of the SPATT-Accumulated Toxins?

The goodness-of-fit, shown by the high percentage of the variance explained (40% to 98%), and the slope of the regression (close to 1) confirm that simulations based on toxin content (pg cell^−1^) estimated from picked cells of each species of *Dinophysis* are much closer to the actual data than those obtained with estimates of toxin content per cell of *Dinophysis* in plankton concentrates (Table 2). In the last case, the percentage of the variance explained is much lower (7% to 90%) and the slope of the regression is always higher than one and in some cases closer to two. These results indicate that in most of the simulations using toxin content from plankton concentrates, the predicted accumulation in SPATT is almost double the observed value. This may indicate that in those situations, the toxin content per cell is grossly overestimated. This may be the case, for example, with the estimated toxin content of 180 pg PTX2 cell^−1^ from early November. We [9] proposed earlier that similar observations indicate that in plankton net-hauls collected in turbulent water-columns—rich in detritus and fecal pellets and with undetectable levels of *Dinophysis*—most of the toxins detected are those adsorbed by organic aggregates. This view is supported by the fact that accumulation of PTX2 in the resins (and presumably in mussels) decreased considerably from 30 October onwards, when the water column was well mixed.

Nevertheless, the use of a constant average value (pg cell^−1^) for the toxin content of *Dinophysis* cells has some inconveniences. We used this average because we could not obtain weekly estimates of toxin content for all the species. We know very little about the balance between production and release of toxins in field populations of *Dinophysis*, and it may well be that each toxin follows a different pattern of production and release in response to changing environmental conditions and their interaction with the physiological status of the population. In addition, we know already from culture experiments that toxin content per cell results from a balance between division rate (that dilutes toxin accumulation per cell) and toxin production rate, resulting in “more toxic” cells when division declines in stationary phases of the population [20,21,22]. For example, in this study, the tiny amount of DTX2 accumulated in the SPATT on 4 September following a peak (3200 cell·L^−1^) of *D. acuta* the previous week is intriguing. In this particular case, simulations based on toxin content in the plankton concentrates were closer to the actual accumulation of DTX2 (Figure 5 and Figure 6). Unfortunately, no data is available on toxin per cell of *D. acuta* on these days because hardly any cells of this species were collected through the shallow (1–3 m) inflow depth of our pump system, although plenty of cells were detected in the 0–5 m and 5–10 m sections of the hose sampler (Figure 3 and Figure 4). The use of vertical (0–15 m) net hauls as a source for picked cells would have solved this problem, but net hauls are a much “dirtier” material than size-fractioned pump concentrates, and picking of individual cells of *Dinophysis* is much harder with the former.

The mathematical equation used for the simulations in this work was based on that used in the model of Blanco [23] to simulate the accumulation of toxins by molluscan shellfish species. Nevertheless, the loss term of the equation was not included because there is no depuration of toxins on the SPATT, *i.e.*, whatever is adsorbed remains accumulated in the discs. In this sense, SPATT cannot mimic the accumulation of toxins by mussels at a given time, because in these bivalves elimination of toxins starts at the very moment they are ingested, mainly in the feces [24]. Therefore, SPATT may accumulate a much higher amount of toxins than those a mussel would be able to during the same period of time. The good fits obtained for most of the toxins and depths where SPATT was deployed (with a model based on the cell density of *Dinophysis* and using a constant value for the toxin content per cell) indicates that what SPATT data basically reflect are variations in cell density and their toxin content, and probably more reliably than is obtained with discrete sampling. This is because SPATT is fixed and continuously exposed to the spatio-temporal variability in *Dinophysis* distributions, thus behaving as an artificial mussel that integrates results over time. The good fits with *Dinophysis*-borne toxins also suggest that variations in the percentage of particulate and dissolved toxins in the real world are not as large as those reported from cultures of *D. acuminata* [21,25], *D. acuta* [22] and of other dinoflagellates producers of lipophilic toxins, such as *Protoceratium reticulatum* and *Lingulodinium polyedra* [26,27].

An important question concerning the PTX2SA is whether the source of this toxin in the SPATT was transformed PTX2, either from broken cells of *Dinophysis* present in the water column or degradation within the SPATT resins themselves. If either option were the case, a lag between fits of the simulations for this toxin and those for OA and DTX2 should be observed, because part of the particulate PTX2 in *Dinophysis* cells would be converted into PTX2SA. Nevertheless the goodness-of-fit in the case of PTX2 simulations with toxin per picked cell data is much better than that for the OA and DTX2 simulations. These results suggest an alternative source for the accumulated PTX2 other than *Dinophysis* cells. Our hypothesis is that the source of PTX2SA accumulated in the SPATT is the release of PTX2SA contained in fecal pellets of mussels and copepods following ingestion of *Dinophysis* cells. Two facts support this hypothesis: (i) The location of our SPATT discs that were deployed on a rope contiguous to the mussel ropes of a mussel cultivation raft; (ii) the arrest of PTX2SA accumulation in the SPATT after 4 December when toxins in mussels dropped below regulatory levels, although OA and PTX2 were accumulated until the end of the study.

### 3.3. Assessment of the Use of SPATT as a Tool in Research and Monitoring of Lipophilic Toxin Outbreaks

**Figure 7:**
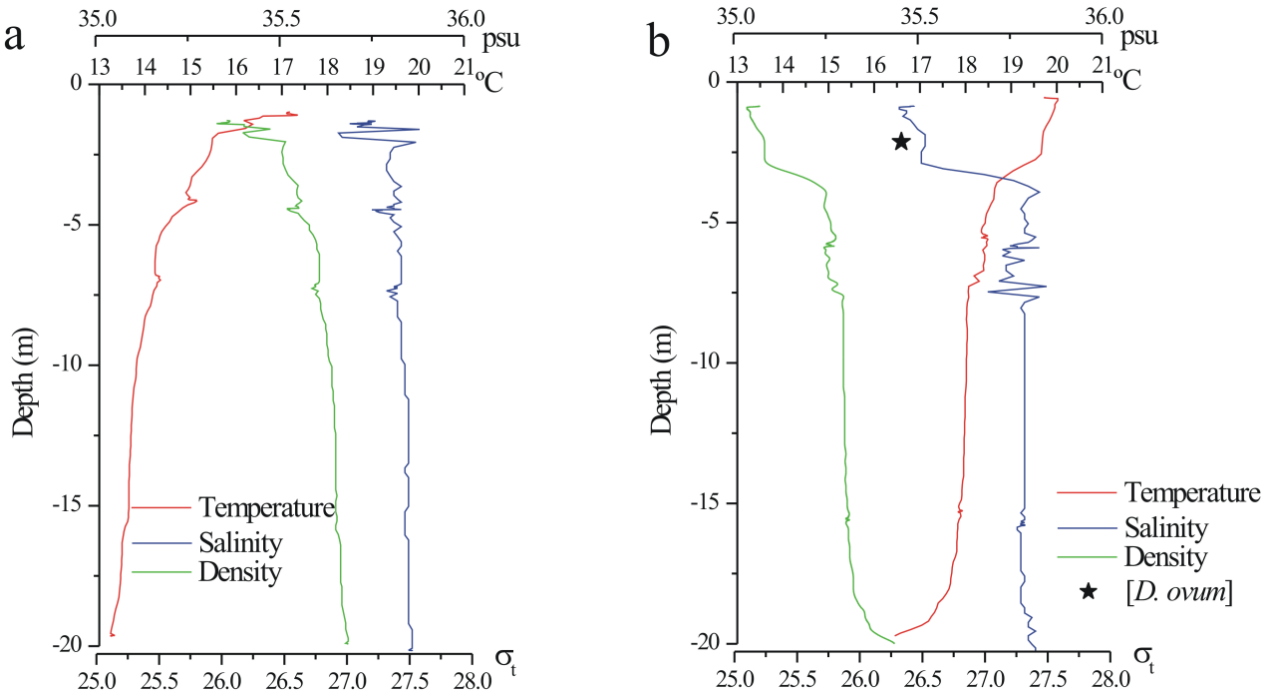
Vertical distribution of temperature (°C), salinity (psu) and density (sigma-t) at the sampling station P2 on (**a**) 5 June (**b**) 12 June 2006. The asterisk marks the depth where the pump sample dominated by *D. ovum* was taken.

Weekly monitoring of harmful algal bloom (HAB) species provides *Dinophysis* densities (from 0–5, 5–10, 10–15, 15–20 m) at a single station and time of day, but the diurnal vertical migration (DVM) of each species combined with short-term variability associated with the local wind-driven circulation and tides may lead to dramatic changes in vertical distribution on a scale of days (wind shifts) and hours (diurnal tidal cycle). In contrast, SPATT discs accumulate toxins from the previous 7 days and integrate vertical distribution variability. Therefore, we cannot always expect good correlations between toxins adsorbed by the resins and co-occurring *Dinophysis* densities. Integrated water column samples also integrate vertical discontinuities. A good example of these discrepancies between integrated *versus* single-depth samples [4] was the almost mono-specific bloom of *D. ovum* detected with the pumped size-fractioned plankton concentrate on 12 June, that in the 0–5 m hose sample co-occurred (50% of each) with *D. acuminata*. The data show that a marked density gradient was formed at about 3 m depth on 12 June when the pump sample was collected, and *D. ovum* was probably aggregated in a thin layer just where the pump intake was cast (Figure 7). Preferential aggregation of *D. ovum* in near surface layers of the water column (where the pump samples were taken) would also explain the dominance of this species until late June in the plankton concentrates, whereas *D. acuminata* was dominant in the monitoring water column samples at 0–5 m. These early peaks of *D. ovum* and *D. acuminata* did not lead to any accumulation of toxins in the SPATT at any depth. Either cells from early stages of the bloom did not release any toxins or they were forming very short-lasting thin layers embedded in a diatom-dominated microplankton community.

From earlier studies [15,19,28] in addition to results presented here, all using the same analytical methods, the positive associations between the accumulation of OA and the peaks of *D*. *acuminata* and *D. acuta*, between DTX2 and the occurrence of *D. acuta* and between PTX2 and both, *D. acuta* and *D. caudata* in Galician coastal waters, seem clear.

The main question to be posed for phycotoxin monitoring programs is: Is the SPATT useful as an early warning tool for diarrhetic shellfish poisoning events? Figure 4a,b shows several examples where detection (≥40 cell·L^−1^) of *Dinophysis* cells took place well ahead (several weeks) of the detection of toxins in the resins. The first detection of OA in the SPATT was one week after the first seasonal increase of *Dinophysis* in surface waters (Figure 4a,b). Further, accumulation of toxins was maximal during late stages of the populations of different species of *Dinophysis*, suggesting that toxin-release is higher during late exponential, early stationary growth phases. Therefore, detection of *Dinophysis* cells, sampled with sufficient frequency (weekly at least) and spatial coverage provides the best early warning of the potential risk of future accumulation of toxins in shellfish above regulatory levels (RL). In the present study, detectable accumulation of OA in the SPATT discs took place 2–3 weeks before concentrations of this toxin in mussels—controlled by mouse bioassays (MBA)—was above RL. Nevertheless, in a control laboratory where lipophilic toxins are routinely monitored with LC-MS analyses of shellfish flesh, toxins in the SPATT would be detected more or less at the same time as in shellfish, as already found in Irish waters [29], or maybe a few days in advance, since accumulation in the resins is sometimes much higher than in shellfish. This is the case when high concentrations of total phytoplankton lead to decreased filtration rates in bivalves [30].

The amount of toxins adsorbed per week by the SPATT during the 2006 maximum of *D. acuta* in the present work was larger than those cited during blooms of the same species in New Zealand [11] and Ireland [12] and about double the maximum amount collected during the 2005 bloom in the same location the previous year [14]. Therefore, the use of SPATT allows an evaluation of the intensity of DSP outbreaks in different regions and of interannual variability in the same location.

Accumulation of toxins in the SPATT lasted more than 1.5 months after the end of shellfish harvesting closures in the case of OA and PTX2, but this was reduced to 1 week in the case of DTX2, a toxin that seems to be much less stable after its release in seawater. We could conclude that resins work as a more sensitive artificial mussel. But resins do not reflect enzymatic changes of the toxin profile that take place within the shellfish flesh. For example, mussels can transform all the ingested PTX2 into PTX2SA, and then be safe for human consumption, while the resins continue to detect PTX2 in the water column. Likewise, SPATT will in most cases overestimate the actual accumulation in shellfish because in bivalves we have to include fecal pellets as a loss factor (see the previous section). We can conclude that SPATT can be useful as a tool for early detection of initiation or advection of *Dinophysis* populations in areas not subject to toxin monitoring but that may represent a source of toxic cells to the aquaculture sites. In that case, they should be deployed in a wide range of depths including near the bottom and in the pycnocline. This kind of application has allowed detection of *Dinophysis* populations and/or their toxins in deep shelf waters in SW Ireland [31].

## 4. Experimental Section

### 4.1. Field Sampling

**Figure 8:**
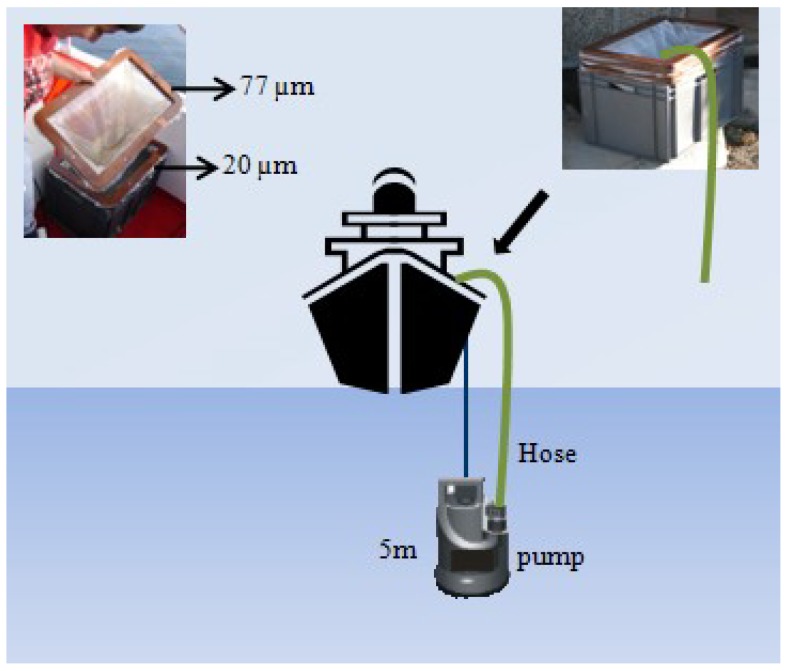
Simplified diagram of the submersible pump and superimposed framed meshes system used to obtain the 77–20 µm size-fractioned plankton concentrates.

Weekly sampling was carried out during 2006, on board R/V *Navaz*, at a fixed station (P2, 42°21.40′N, 8°46.42′W) in Ría de Pontevedra (Figure 1), a hot spot for DSP outbreaks according to historic data from monitoring programs in the region. Phytoplankton samples for toxin analyses were collected from 5 June—when the presence of *Dinophysis* spp. started to be reported by the Galician monitoring centre (INTECMAR) (www.intecmar.es)—until 18 December 2006. Plankton 20–77 µm size-fractioned concentrates were obtained by pumping seawater with a submersible pump (flow of 138 L min^−1^) from a fixed depth in the top 5 m of the water column for 5–10 min, through a set of superimposed screens with meshes of decreasing size (100, 77 and 20 μm). The screens were set over a container with a drain-overflow located at the top, above the base level of the nets, so that the plankton concentrate was always kept submerged (Figure 8). This concentrate was re-suspended in 5-L bottles of seawater to keep the cells in healthy conditions until arrival at the laboratory (approximately 1 h). A 50-mL subsample of this “pump-concentrate” was preserved with Lugol’s iodine solution for cell identification and quantification. Density (cells·L^−1^) and relative abundance of *Dinophysis* species in these pump-concentrates from the 3–5 m water layer were not expected to represent the population in the whole water column. They were obtained to collect enough cells of *Dinophysis* for toxin analyses (toxin profile and content) of individually picked cells of each species and of the total microplankton community.

**Figure 9:**
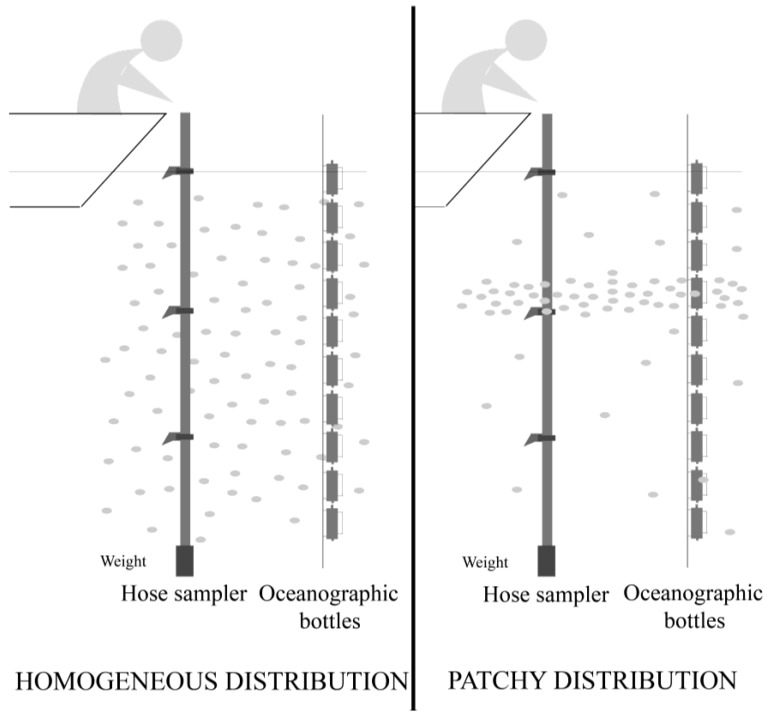
Simplified diagram of a dividable tube (hose) sampler and sources of discrepancies between integrated water column (hose sampler [32]) and specific depth (oceanographic bottles) samples when phytoplankton cells are aggregated in thin layers (right). Reproduced with permission from [4], Copyright © 2012 Elsevier.

Simultaneously, integrated water column samples for plankton analyses were collected at the same station with a dividable hose sampler (0–5, 5–10, 10–15 m), as described by Lindahl [32], by the Galician Monitoring Centre (www.intecmar.org) and immediately fixed with Lugol’s solution (Figure 9).

DIAION HP20 adsorbing resins filling the SPATT discs were used to track extracellular lipophilic toxins in the water column at 7-day intervals between 20 February and 18 December 2006. Activation was performed, as described in MacKenzie *et al*. [11] by leaving the DIAION HP20 resins (100 g) in contact with methanol (1 L) overnight, prior to filtration through a 23-µm mesh, resuspension in 1 L of MQ water for 30 min, drainage and storage in 1 L MilliQ water at 4 °C until use.

SPATT discs made with 77-μm plankton mesh mounted on embroidery hoops (9-cm diameter), and filled with 25 mL of a suspension of resins in Milli-Q water (equivalent to 2.5 g dry weight of resin), were deployed at 3, 7 and 12 m depth on a rope hung from a mussel raft. Each time, SPATT discs from the previous week were collected and replaced by new ones in the same positions. Care was taken that the resin-holders did not dry out before being placed in the water. The possibility of resin saturation in the field was discarded after testing in an earlier study [9].

### 4.2. Phytoplankton Counts

For identification and quantification of *Dinophysis* species and other potential toxin producers, aliquots of the Lugol-fixed pump-concentrate samples were placed in 3-mL HydroBios sedimentation chambers (Hydro-Bios Apparatebau GmbH, Kiel, Holtenau, Germany), left to settle for a few hours, and the whole surface of the chamber scanned at a magnification of 100× under a Nikon Eclipse TE2000-S inverted microscope (Nikon, Tokyo, Japan). To estimate toxin content per cell in the pump-concentrates, the total amount of toxins estimated from the 15 mL extract (see Section 3.3) was divided between the number of *Dinophysis* cells contained in that volume. Phytoplankton analyses at INTECMAR were carried out according to Utermöhl [33]. Aliquots of 25 mL of the 0–5 m, 5–10 m, 10–15 m and 15–20 m integrated water column samples were left to sediment overnight. To enumerate large species, such as *Dinophysis* spp., the whole surface of the chamber was scanned at a magnification of 100× under the inverted microscope (detection level of 40 cell·L^−1^).

### 4.3. Toxin Extraction from Phytoplankton Pump-Concentrates, Picked Cells and SPATT Resins

In the laboratory, 15 mL aliquots of the 20–77 μm fraction of phytoplankton concentrates were centrifuged for 20 min at 1000 rpm and the supernatants eliminated; pellets were re-suspended in 500 μL of methanol, the mixture sonicated for 1 min (35–40 KHz) and the samples kept frozen until analysis. Just before analyses, samples were defrosted, centrifuged at 5000 rpm for 20 min, the supernatant collected, and the pellet re-suspended with 2 mL of methanol for a second extraction repeating the same procedure. The two supernatants were combined, mixed and methanol added to make up a final volume of 4 mL. A 1.5-mL aliquot of this solution was dried at 40 °C under reduced pressure on a Speed Vac (Savant, Instruments Inc., Holbrook, NY, USA), re-suspended in 150 µL of methanol and filtered through 0.45-µm filters (13-mm Gelman Nylon Acrodisc, Albany, NY, USA or 3-mm Osmonic Inc., Cameo 3N, Minnetonka, MN, USA) prior to injection into the LC-MS system.

*Dinophysis* cells were individually picked from the 20–77 μm fraction of plankton concentrates with a microcapillary pipette under the inverted microscope (40× and 100×), and passed two to three times through filtered (0.22 μm) seawater. Between 30 and 100 cells of each species were transferred to 1.5-mL Eppendorf tubes filled with 250 µL mL of Tris–HCl buffer (50 mM, pH 7.4); 250 µL of methanol were added and the mixture sonicated for 1 min. The final extract-samples—kept in the deep freezer until analysis—were transferred to vials of 1.8 mL, the remains in the Eppendorf tube washed with 500 µL of methanol and the supernatants combined and mixed. This solution was dried and processed in the same way as those obtained from plankton-concentrates.

Extraction of toxins from the SPATT discs was carried out following Pizarro *et al*. [14]. Briefly, after 7 days of exposure, SPATT discs were recovered and the resins washed with Milli-Q water (40 mL) to eliminate sea salts. After drainage, the resin was soaked with 20 mL of methanol and left a minimum of 2 h before elution of the methanol. The resin was then washed five times with 10 mL of methanol (flow of 0.5 mL min^−1^) until a final volume of 50 mL was reached. These pre-extraction samples were kept below 4 °C until analysis. To prepare for the LC-MS analysis, 4 mL of the extract were dried at 40 °C under reduced pressure on a vacuum concentrator (Speed Vac), resuspended in 500 µL of methanol and filtered through 0.45-µm filters prior to injection into the LC-MS system.

### 4.4. LC-MS Analyses

LC-MS analyses were performed on a Thermo Finnigan Surveyor coupled to an ion-trap mass spectrometer (Thermo Finnigan LCQ-Advantage) equipped with a micro-electrospray ionization interface (μESI). Separations were achieved on a Waters XBridge C18 5-μm (2.1 × 150 mm) column at 35 °C. The mobile phase consisted of 2 mM ammonium acetate at pH 5.8 (A) and methanol (B). A linear gradient elution of 60%–100% B for 20 min followed by 100% B for 2 min and re-equilibration with 60% B for 8 min was used. The flow rate was 0.2 mL min^−1^. The sample injection volume was variable (5–20 µL) depending on the toxin concentration in the sample. μESI was performed at a capillary temperature of 250 °C and a spray voltage of 3.0 and 4.5 kV for positive and negative analyses, respectively. Flows of 20 mL min^−1^ for sheath gas and of 10 mL min^−1^ for auxiliary gas were used. Full scan data were acquired from *m/z* 300 to 2000, in both negative and positive ionization modes. Identification of the toxins OA, DTX2, PTX2 and PTX2 SA was performed by LC-MS/MS in an earlier work by applying a supplementary voltage (Collision Energy, CE) of 60 eV on the precursor ions at *m/z* [M + NH_4_]^+^ [9]. Once these toxins were identified, routine analyses were performed by LC-MS for the sake of cost and rapidity of the analyses. The LC-MS analyses were carried out using the ions at *m/z* 805 [M + H]^+^, 822 [M + NH_4_]^+^, 827 [M + Na]^+^ and 803 [M − H]^−^ for OA and DTX2; 876 [M + NH_4_]^+^, 881 [M + Na]^+^, 857 [M − H]^−^ and 918 [M + CH_3_COO]^−^ for PTX2; 894 [M + NH_4_]^+^, 899 [M + Na]^+^ and 875 [M − H]^−^ for PTX2SA. Predominant ions were detected in positive mode for OA, DTX2, PTX2 and PTX2SA. Negative mode was performed only to confirm the toxin spectrum.

In the case of picked cells, to obtain a discernible signal according to the possibilities of the mass spectrometer, a single ion monitoring (SIM) analysis and positive ionization mode alone were used to record signals of the [M + NH_4_]^+^ and [M + Na]^+^ ions for OA, DTX2 and PTX2. The presence of PTX2SA—considered an enzymatic transformation of PTX2 due to poor handling of plankton samples—was not explored as it has never been detected in phytoplankton samples in previous studies in the region [9,14].

For the analyses of plankton concentrates and SPATT extracts, working solutions of OA, DTX2 and PTX2—0.6 ng µL^−1^ approximately, detection limit (DL) 6 ng µL^−1^—and PTX2SA—0.3 ng µL^−1^ approximately, DL 3 ng µL^−1^—were used to generate a four-point calibration curve with variable injection volumes. Concentration ranges for the calibration lines obtained for each toxin were from 1.5 to 6.0 ng for OA and DTX2, from 1.8 to 7.0 ng for PTX2 and from 0.5 to 3.0 ng for PTX2SA. For picked cells, a working solution of OA and DTX2—4 pg µL^−1^ approximately, DL 40 pg µL^−1^—was used from 8 to 40 pg. A four-point calibration curve was generated with variable injection volumes. The linearity of each point was tested following van Trijp and Roos [34]. Full details of the LC-MS chromatograms and mass spectra in positive ionization mode are given in Pizarro *et al*. [9].

### 4.5. Simulation of Toxins Adsorption by the SPATT Discs

The adsorption of each type of toxin (OA, DTX2, PTX2) by the SPATT resins was mathematically simulated considering that SPATT at each depth behaved as an artificial mussel—with an adsorption rate of 3 L h^−1^—which accumulated all the particulate toxins available in the filtered seawater following the equation:

*dTa/dt* = *C*_cell_*C*_tox_*F*_ads_ (1)

where *Ta* is the amount of toxin adsorbed by the SPATT; *C*_cell_ is the *Dinophysis* cell concentration per liter of seawater; *C*_tox_ is the pg of toxin per cell of *Dinophysis* and *F*_ads_ is the rate of toxin adsorption by the SPATT that was given a constant value of 3 L h^−1^.

Two kinds of simulations were made for each type of toxin (OA, DTX2, PTX2) and depth (3 m, 7 m, 12 m) of SPATT deployment: (i) one simulation that used the average estimate of toxin content (pg cell^−1^) per cell, for each species of *Dinophysis* used in the model, from the analyses of picked cells; (ii) another simulation that used the estimate of toxin content (pg cell^−1^) per cell of *Dinophysis* (daily interpolation between two consecutive weekly values) obtained from the analyses of the size-fractioned (77–20 µm) plankton concentrates.

Data of *Dinophysis* cells per liter were interpolated daily between two consecutive weekly values. *Dinophysis* species included in the simulations for each kind of toxin were: (i) *Dinophysis acuminata* + *Dinophysis acuta* for OA; (ii) *Dinophysis acuta* for DTX2 and (iii) *Dinophysis acuta* + *Dinophysis caudata* for PTX2.

## 5. Conclusions

A systematic monitoring of HABs and lipophilic toxins (LC-MS) in picked cells of *Dinophysis* and in plankton concentrates led to confirmation of *D. acuminata* and *D. acuta* as the main sources of OA in Galician shellfish, *D. acuta* of DTX2 and both *D. acuta* and *D. caudata* of PTX2. Discrepancies between toxin per cell estimates and those from plankton concentrates suggest increased amounts of released toxins in the water during late stages of the bloom, which are attached to organic aggregates retained in the filters and not taken up by mussels. Likewise, discrepancies between toxins in plankton concentrates and those accumulated by the SPATT suggest zooplankton and shellfish faecal pellets as the source of PTX2SA in the resins. Model simulations showed best-fit between weekly toxin accumulation by the SPATT discs and the toxin content of *Dinophysis* cells per volume of water, estimated from a constant value of toxin content for each species, averaged from LC-MS analyses of picked cells. OA and PTX2 can persist “dissolved” in the water for weeks but DTX2 is less stable. Monitoring of *Dinophysis* cells with appropriate spatial (integrated samples) and temporal frequency provides earlier warning of DSP outbreaks than SPATT resins, which allow detection of toxins with a similar timing than that of detection in shellfish tissues by LC-MS analyses in regulatory centers. In addition, SPATT accumulation of toxins overestimates that taking place in shellfish and subject to loss terms (enzymatic transformation, fecal pellet elimination); further, SPATT continues accumulating toxins for weeks after *Dinophysis* cells become undetectable and mussels are depurated. SPATT provides a valuable tool for the study of *Dinophysis* population and their toxin dynamics, in particular if deployed in areas and depths not subject to regular surveillance. Further, it has the potential to be a relatively simple method which may provide a more efficient regulatory action, cost-savings, enhanced environmental toxin detection and a monitoring perspective that is less susceptible to the variability of discrete phytoplankton and shellfish sample collections. However, SPATT does not represent a practical gain for early warning of DSP outbreaks in aquaculture sites subject to routine monitoring of HABs and phycotoxins and for the time being should remain as a complementary tool to traditional sampling.
